# An Integrative Engagement Model of Digital Psychotherapy: Exploratory Focus Group Findings

**DOI:** 10.2196/41428

**Published:** 2023-04-26

**Authors:** James M Zech, Morgan Johnson, Michael D Pullmann, Thomas D Hull, Tim Althoff, Sean A Munson, Nicole Fridling, Boris Litvin, Jerilyn Wu, Patricia A Areán

**Affiliations:** 1 Talkspace New York, NY United States; 2 Department of Psychology Florida State University Tallahassee, FL United States; 3 Department of Psychiatry & Behavioral Sciences University of Washington Seattle, WA United States; 4 Allen School of Computer Science & Engineering University of Washington Seattle, WA United States; 5 Department of Human Centered Design & Engineering University of Washington Seattle, WA United States

**Keywords:** Health Action Process Approach, lived informatics, digital engagement, messaging therapy

## Abstract

**Background:**

Digital mental health interventions, such as 2-way and asynchronous messaging therapy, are a growing part of the mental health care treatment ecosystem, yet little is known about how users engage with these interventions over the course of their treatment journeys. User engagement, or client behaviors and therapeutic relationships that facilitate positive treatment outcomes, is a necessary condition for the effectiveness of any digital treatment. Developing a better understanding of the factors that impact user engagement can impact the overall effectiveness of digital psychotherapy. Mapping the user experience in digital therapy may be facilitated by integrating theories from several fields. Specifically, health science’s Health Action Process Approach and human-computer interaction’s Lived Informatics Model may be usefully synthesized with relational constructs from psychotherapy process–outcome research to identify the determinants of engagement in digital messaging therapy.

**Objective:**

This study aims to capture insights into digital therapy users’ engagement patterns through a qualitative analysis of focus group sessions. We aimed to synthesize emergent intrapersonal and relational determinants of engagement into an integrative framework of engagement in digital therapy.

**Methods:**

A total of 24 focus group participants were recruited to participate in 1 of 5 synchronous focus group sessions held between October and November 2021. Participant responses were coded by 2 researchers using thematic analysis.

**Results:**

Coders identified 10 relevant constructs and 24 subconstructs that can collectively account for users’ engagement and experience trajectories in the context of digital therapy. Although users’ engagement trajectories in digital therapy varied widely, they were principally informed by intrapsychic factors (eg, self-efficacy and outcome expectancy), interpersonal factors (eg, the therapeutic alliance and its rupture), and external factors (eg, treatment costs and social support). These constructs were organized into a proposed Integrative Engagement Model of Digital Psychotherapy. Notably, every participant in the focus groups indicated that their ability to connect with their therapist was among the most important factors that were considered in continuing or terminating treatment.

**Conclusions:**

Engagement in messaging therapy may be usefully approached through an interdisciplinary lens, linking constructs from health science, human-computer interaction studies, and clinical science in an integrative engagement framework. Taken together, our results suggest that users may not view the digital psychotherapy platform itself as a treatment so much as a means of gaining access to a helping provider, that is, users did not see themselves as engaging with a platform but instead viewed their experience as a healing relationship. The findings of this study suggest that a better understanding of user engagement is crucial for enhancing the effectiveness of digital mental health interventions, and future research should continue to explore the underlying factors that contribute to engagement in digital mental health interventions.

**Trial Registration:**

ClinicalTrials.gov NCT04507360; https://clinicaltrials.gov/ct2/show/NCT04507360

## Introduction

### Background

Digital technologies are a burgeoning research area in clinical science and are an increasingly substantial part of modern mental health services. Owing to their relative scalability and cost-effectiveness versus in-person care, the global market for digital mental health (DMH) applications—devices and programs that use technology to facilitate behavior change and mental health [1,2]—is expected to reach US $10.2 billion by 2027 [3]. Beyond their growing market share, DMH interventions have been found to be widely acceptable and clinically efficacious in treating a range of mental health issues, including anxiety disorders [4], depression [5], obsessive-compulsive disorder [6], substance dependence [7], and behavioral addictions [8]. These DMH interventions take a wide variety of forms, from unguided self-help mobile apps to peer support platforms and digitally enabled message- and video-based psychotherapy. Wholly self-guided interventions incur near-zero marginal administration costs and are therefore easier to widely disseminate than peer- or clinician-guided interventions. However, prior research has found that guided interventions achieve better clinical outcomes than unguided interventions [9], which may be linked to higher rates of user engagement and treatment adherence in guided protocols.

Although efficacious, guided DMH interventions exist, they can only be clinically effective insofar as they elicit appropriate levels of user engagement. As the DMH research and product landscape has matured, consistent engagement has been identified as the sine qua non of digital care and a key area for future research [10]. But “engagement” itself is not an easily defined concept. In conventional mental and behavioral health research, engagement has been typically defined as a multidomain construct that consists of client behaviors (eg, attendance and participation in therapy sessions and homework completion), client attitudes (eg, expectations about treatment and one’s readiness), and client-therapist relationships (eg, therapeutic or working alliance) that facilitate positive treatment outcomes [11,12]. Sometimes, other engagement domains have been described, such as client empowerment, involvement of social networks in treatment, and client understanding of the treatment approach and therapy roles, but behaviors, attitudes, and relationships are the most frequently operationalized.

Specific to DMH, several promising conceptualizations exist [13-15] but the DMH research community presently has no widely agreed-upon definition of user engagement or explanation of the mechanisms by which engagement affects behavior change. Indeed, a 2019 review of engagement in human-computer interaction identified a total of 102 definitions for “engagement” spanning 351 articles [16]. This lack of conceptual consensus may be due, in part, to a lack of guiding theory. Research on digital engagement has been criticized for not being guided by underlying theories of behavior change [17]. This diversity of definitions and lack of conceptual clarity are understandable, considering that different digital interventions offer different features that promote different engagement patterns. Yet, at base, any psychosocial mental health treatment involves collaboration on the part of the patient and provider to engage in circumscribed therapeutic acts, which may differ greatly based on the psychological issues being treated and the therapeutic modality applied [18], that is, for any treatment to be effective, there must be some level of user engagement in the therapeutic tasks involved therein (eg, self-monitoring, self-disclosure, doing homework, or exploring difficult emotional topics with a provider). In this study, we focused primarily on engagement, as indicated by client behaviors (eg, use of DMH to engage in therapeutic tasks) and therapeutic relationships (eg, use of DMH to achieve a bond between clients and therapists primarily characterized by a shared understanding of and commitment to achieving therapeutic tasks and goals) that facilitate positive treatment outcomes. For instance, a DMH tool that facilitates the use of texting between therapists and clients may operationalize engagement as the number of texts sent, words per text, the valence and therapeutic content of the words, and the degree to which the client feels the DMH tool facilitates therapeutic alliance. A DMH tool for self-monitoring thoughts and behaviors might operationalize engagement as the amount of time the user spends on the app, the number and types of thoughts and behaviors being monitored, and the use of app-provided cognitive tools for addressing undesired thoughts and behaviors.

Prior empirical research suggests that user engagement may be an important determinant of clinical outcomes in DMH [19]. In a recent observational study [20] of a digital psychotherapy platform [21], of 10,718 users with clinically elevated levels of depression or anxiety, 67.6% met full recovery criteria, with user engagement defined by words sent by the user substantially predicting better response trajectories. This meaningful association between engagement and clinical outcomes was replicated in a subsequent study on the treatment of posttraumatic stress disorder [22]. However, research has found that engagement in DMH [23], such as engagement in traditional psychotherapy, is poor. Less than 30% of self-guided DMH consumers use services for 90 days, and the average engagement is 2 to 4 weeks, with most consumers discontinuing self-guided apps within hours of initial use [24-26]. When DMH is supported by coaches or clinicians, engagement is somewhat better, but long-term engagement remains a challenge. Less than 1% of consumers who access clinician-assisted DMH complete all sessions [27]. Our research on text-based therapy found that over a third of clients disengaged within 6 weeks of initiating treatment [28], with disengagement rates higher for clients who were younger, had higher education, and had been in therapy before. Aside from these simplistic demographic determinants, we know very little about why a client might engage in DMH and how to tailor treatment to ensure optimal engagement. By grounding DMH research in existing theories of behavior change, it may be possible to arrive at a clearer understanding of the intrapersonal and contextual factors that influence user engagement.

### The Health Action Process Approach and the Lived Informatics Model

**Figure 1:**
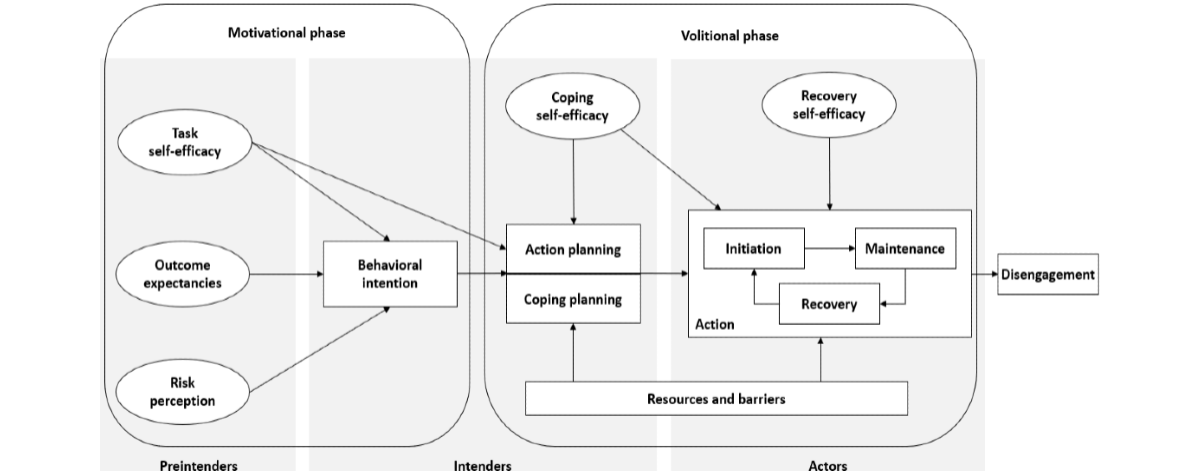
The Health Action Process Approach describes a multiphasic approach to behavior change.

There are a number of behavior change frameworks that could be usefully applied to the study of DMH interventions, but 1 model, The Health Action Process Approach (HAPA) [29,30] (Figure 1), is distinguished by its considerable empirical support in other health behavior change contexts. The HAPA model, originally developed as a synthesis of social cognitive theory [31] and the theory of reasoned action [32], describes behavior change as a multiphasic process. Per HAPA, a “preintentional” individual first forms an intention to make some behavior change during an initial motivational phase. This intention formation is mediated by the individual’s perceived self-efficacy, net outcome expectancies attendant to engaging in health behavior, and risk perceptions related to the negative consequences of abstaining from the behavior change. Once a behavioral intention forms, the individual plans the specific steps required to engage in the desired behavior in the planning phase, engage in the behavior in the action phase, and work to continue that behavior in the maintenance phase.

The core constructs of the HAPA model have been empirically tested in at least 95 studies [30]. Core HAPA constructs such as self-efficacy and outcome expectancy have been shown to be associated with positive health outcomes in the context of smoking cessation [33], physical activity [34,35], nutrition management protocols [36], trauma recovery [37], and mindfulness training for general mental health promotion [38]. However, recent meta-analytic findings also indicate that not all putative HAPA mediators and moderators are equally relevant across contexts [30]. Notably, there is relatively little empirical support for the mediating role of risk perception in behavioral intention formation versus other core intrapersonal constructs. These researchers also found that behavioral intention had a smaller effect on subsequent health behaviors in clinical populations. These findings not only suggest that the HAPA framework may be a valid conceptual starting point but also suggest that theorists must independently validate this model before applying it to novel contexts such as DMH treatments.

Although the HAPA framework could support conceptualizing DMH engagement, it has at least 1 key limitation: it is a primarily intrapersonal model of health promotion, focusing principally on intrapsychic variables (eg, one’s beliefs, motivations, and volitions) as mediators of desired outcomes. However, psychotherapy, including digitally enabled psychotherapy, is a fundamentally interpersonal process of change involving 2 principal actors: a patient and a provider. Similarly, some of the most well-supported psychotherapeutic mediators of change have also been fundamentally interpersonal. One notable example is the therapeutic alliance—a tripartite construct involving the development of an authentic bond between the client and provider, as well as an agreement on the desired goals of therapy and the tasks required by both parties to affect these goals [39,40]. One of the most consistent findings to emerge from psychotherapy process–outcome research is that the therapeutic alliance is strongly predictive of clinical outcomes [41]. The strength of the alliance is not always stable over time and is liable to “rupture” over the course of therapy [42,43]. Early work in DMH suggests that there is no difference in client ratings of therapeutic alliance in digital versus in-person contexts [44], although recent reviews on the study of therapeutic alliance in DMH note the need for more rigorous empirical testing of this putative process-outcome relationship [45]. Given the apparent centrality of the therapeutic alliance in psychotherapy, it is reasonable to posit that this and related constructs can, similar to the HAPA framework, be usefully leveraged to better understand the determinants of DMH engagement.

**Figure 2:**
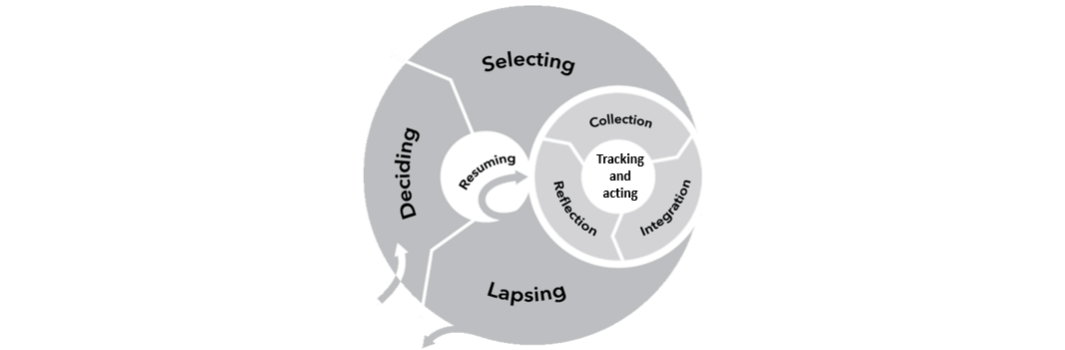
The Lived Informatics Model emphasizes the importance of deciding to engage in an intervention, selecting tools, disengagement, and resumption alongside the activities within a particular intervention.

A further limitation of the HAPA model and other behavior change theories is that it does not account for the way that users may engage, disengage, and reengage with DMH interventions over the course of their user experience. These variable engagement patterns may be due to a range of factors, such as changing personal goals or contextual changes (eg, vacation). To account for variable and cyclical engagement patterns across the user life cycle, we drew on the Lived Informatics Model (LIM) [46]. Although developed based on engagement with and the use of personal data tools, its overall framework can be applied to describe engagement with a range of DMH interventions. According to the LIM, as people progress toward their initial goals, those goals evolve, which then leads to differing engagement patterns with DMH tools (Figure 2). Accordingly, the LIM rejects the notion of ongoing, stable engagement and highlights the importance of designing for both intended lapses (eg, choosing to take a break from the intervention during a vacation or a busy time at work) and unintended lapses (eg, forgetting to use the intervention until engagement ceases, at least temporarily, and having internet connectivity problems that prevent the use of the intervention for a time) and resumption from those lapses. In addition, the model emphasizes the importance of tool selection, both initially and by revisiting the choice of tools as one’s knowledge and goals progress [47].

### Study Design and Objectives

In this study, we sought to explore the determinants of user engagement patterns by conducting qualitative focus groups with users of a digital therapy platform [21]. Focus groups were chosen as our primary data collection method because this study was exploratory and theory building. Focus groups allow for rich and detailed qualitative data to be collected not only through participants’ initial responses but also through group interaction and discussion [48,49]. Participants can build on each other’s ideas and perspectives, generating a more in-depth understanding of the research topic. This is particularly relevant in the present context, where engagement in digital treatment is likely to be driven by multiple intersecting factors and can be particularly useful in identifying subtle differences that may exist based on factors such as age, gender, or diagnostic background. In addition, focus groups are a highly efficient method of collecting qualitative data compared with arranging individual interviews with each participant. In analyzing this study’s focus group findings, we synthesized HAPA constructs with both theories of psychotherapeutic process and the LIM to conceptualize the key factors determining user experiences and DMH platform engagement patterns, which we present as an Integrative Engagement (IE) Model of Digital Psychotherapy.

## Methods

### Setting

The platform [21] facilitates the pairing of treatment seekers with independently licensed therapists and provides several media types for the delivery of care: daily messaging only, messaging plus monthly video sessions, messaging plus weekly video sessions, and telepsychiatry. Regardless of the therapeutic modality chosen, each user is offered an introductory live video session with their provider. Therapists are predominantly masters-level clinicians, with an average of 9 years of postlicensure experience. Users can self-pay for the service with a monthly subscription or use it as part of their employment or health plan benefits. Following a standardized intake, individuals are matched with a clinician according to their preferences and diagnostic criteria within 24 to 48 hours. Clinicians provide informed consent, discuss the frame of the relevant medium, and perform diagnostic interviews, after which treatment unfolds in collaboration with the patient and according to the clinician’s judgment. Referrals are provided for individuals who require a higher level of care or who exhaust their employee or health plan benefits and would like to continue. Users can switch therapists at any time using a self-service feature that restarts the matching process. Therapists can draft and reuse their own scripts (ie, “canned messages”) for routine procedures such as introducing themselves, describing the treatment frame, and obtaining informed consent. However, all other therapist messages are strongly encouraged to be individualized and patient specific. Outcome measures will be deployed at baseline and every 3 weeks for quality management, treatment planning by the therapist, and naturalistic research. The interface options include mobile devices and desktop computers, with the messaging feature enabled for audio and video recordings, as well as image sharing.

### Ethics Approval, Informed Consent, and Participation

This study was approved by the University of Washington Institutional Review Board (STUDY00010958) and conducted in accordance with the ethical principles of the Declaration of Helsinki. All participants provided written informed consent before participating in the study. The participants were drawn from a sample of Talkspace platform users. We recruited participants by emailing a subset of both past and present users who had used the platform at any time between 2012 and 2021. The email invitation contained a description of the study’s purpose, criteria for participation, and information on compensation. Participants were informed that their participation was voluntary and that they could withdraw from the study at any time without penalty. Participants provided informed consent and an auxiliary demographic questionnaire, attended a 90-minute focus group session, and were compensated with a US $40 Amazon gift card for their participation. All data collected were deidentified and kept confidential. We took several measures to ensure the privacy and confidentiality of participants, including using a secure survey platform, encrypting the audio recordings, and storing the data on a password-protected computer. We also ensured that the participants’ responses were deidentified before all analyses.

### Procedure

Five focus group discussions were conducted between October 25 and November 4, 2021, each of which included 2 to 9 participants with prior experience using the platform. All focus groups were held, recorded, and transcribed automatically using a secure Zoom meeting. Participants were asked to briefly introduce themselves and were guided through a series of open-ended discussion questions related to their experience on the platform. Three researchers (JMZ, JW, and TDH) conducted the focus group sessions, ensuring that each participant answered each question while leaving time for open-ended explorations of discussion topics that arose organically. The discussion questions were formulated to uncover the mediating role of core HAPA constructs on engagement. For example, to ascertain participants’ initial outcome expectancies, participants were asked: “When you first signed up, how confident were you that Talkspace would be able to help you?” To understand participants’ risk perception regarding platform use, they were asked: “What concerns or doubts did you have about Talkspace when you first signed up?” In addition, we drew on a model for engagement in technology interventions—the LIM, which focuses on lapsing and resumption as part of the engagement cycle [46]—to ask participants about stopping or pausing and resuming their use of the platform.

Verbatim transcripts were generated through Zoom, and 3 researchers (NF, BL, and JMZ) independently read the transcripts and used these data to summarize each participant’s contribution to the conversation in the form of a narrative vignette. Vignettes were reviewed by all 3 researchers to ensure that all salient information provided by the participants was presented. Each vignette contained approximately 450 words. The vignettes used pseudonyms to ensure participant privacy. Initially, the vignettes were coded by 2 researchers (JMZ and MJ) using a directed coding approach [38], where the constructs of the original HAPA model and LIM were used as the codebook. Although the vignettes were used to simplify the analyses, coders frequently referenced the source transcripts to clarify or expand on information. Although this directed coding approach negated the possibility of grounded theory, the coding team was open to new discovery and theory adaptation and integration based on the fit of emerging codes with the HAPA model and LIM. Several themes (ie, therapeutic alliance) were not represented in the initial codebook; we altered the coding approach to incorporate a semidirected content analysis to supplement the HAPA model constructs with codes that we developed during our analysis. A constant comparative method was used to determine whether additional codes or applications of other existing models were required to explain the data. This data distillation and coding procedure ensured that all relevant concepts were included in the formal coding process. Finally, another round of independent coding ensured that the newly developed coding categories provided a sufficient summary of participant experiences with the platform. A third researcher (MDP) settled disagreements when consensus was not met between the 2 original coders.

### Participants

Table 1 details the demographic characteristics of our focus group participants (N=24). The majority (16/24, 66%) of participants were non-Latinx White (21/24, 87%) and female (18/24, 75%). The plurality of users fell between the ages of 26 and 35 years, although there was a broad range of ages represented. The youngest participant was aged 19 years and the oldest was aged 69 years. Although the demographics of our focus group participants largely mirrored those of the wider platform user base, the proportion of focus group participants with prior therapy experience was relatively small—just 66% (16/24).

**Table 1:** Descriptive statistics of focus group participants (N=24) compared with the Talkspace client population.

			Sample, n (%)		Talkspace (%)
**Ethnicity**					
	White	16 (66)		67.8	
	Asian	3 (12)		8.4	
	Black or African American	3 (12)		13.4	
	≥1 race	2 (8)		0.8	
	Hispanic or Latinx	3 (12)		6.9	
	Not Hispanic or Latinx	21 (87)		93.1	
**Gender**					
	Women	18 (75)		72.1	
	Men	5 (20)		26.1	
	Nonbinary	1 (4)		1.8	
**Age groups (years)**					
	18-25	3 (12)		9	
	26-35	10 (41)		48	
	36-45	3 (12)		29.5	
	46-55	3 (12)		9.3	
	≥56	1 (4)		4.3	
	Not disclosed	4 (16)		N/A^a^	
**Prior experience in therapy**					
	Yes	4 (16)		57.5	
	No	20 (83)		42.5	

^a^N/A: not applicable.

## Results

### Overview

Coders identified 10 constructs, which were segmented into 24 constitutive subconstructs, as shown in Table 2. A portion of these constructs and subconstructs were derived from the original HAPA framework, namely, outcome expectancies, treatment attitudes, self-efficacy, resources and barriers, and action planning. However, the coders encountered 2 difficulties in applying the baseline HAPA frameworks to the focus group discussions. First, there were many instances where HAPA constructs could be either further broken down into subconstructs (eg, the “action planning” stage was composed of 3 questions: “What digital platform should I use?” “What treatment should I seek?” “Which provider should I pair with?”) or truncated into a single construct (eg, merging task self-efficacy, maintenance self-efficacy, and recovery self-efficacy into the single construct self-efficacy). Second, focus group participants discussed topics that, while central to their DMH journey, could not be readily distilled into the HAPA or LIM frameworks; in particular, participants focused on their relationship with their provider. Therefore, the coding team included constructs from the wider psychotherapy process–outcome research literature: therapeutic alliance [39] and therapeutic ruptures [42].

**Figure 3:**
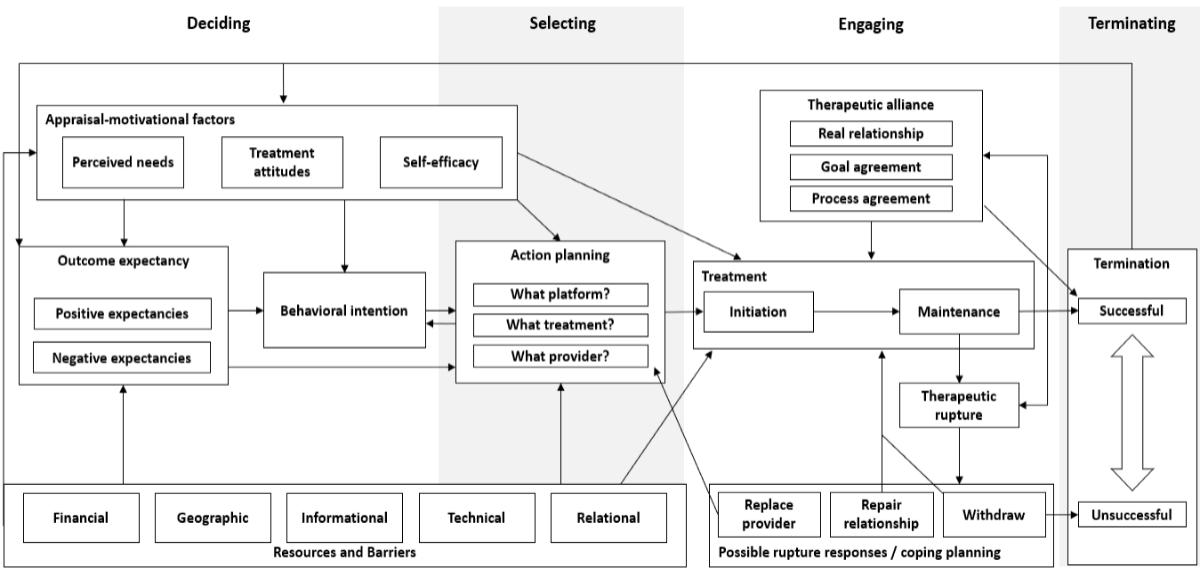
The Integrative Engagement Model.

Over the course of our multi-iterative coding procedure, we developed a new working model of DMH treatment seeking and engagement that integrated HAPA and LIM constructs as well as constructs from the psychotherapy process–outcome literature. This IE Model is illustrated in Figure 3.

**Table 2:** Focus group constructs and subconstructs.

Construct and subconstruct		Example
**Outcome expectancies**		
	Treatment positives	Participant had high expectations when she joined, due to prior experiences with in-person therapy.
	Treatment negatives	Participant was concerned about being heard the same way as in traditional therapy, as there would be no body language.
**Appraisal-motivational factors**		
	Perceived needs	Participant who had several prior suicide attempts understood that foregoing mental health treatment was dangerous.
	Treatment attitudes	Participant began looking forward to her session more and more as treatment progressed.
	Self-efficacy	Participant did not think they could be present during video therapy due to self-consciousness.
Behavioral intention		Participant who had been struggling with mental health issues decided to give treatment a try as a New Year’s resolution.
**Resources and barriers**		
	Financial	Participant’s workplace heavily subsidized his therapy.
	Geographic	Participant lives in a remote part of Washington state, where there were no in-person therapy options.
	Technical	Participant struggled with video sessions on the platform as internet on both ends plays a large part in the success of the call.
	Informational	Participant who thought she would have preferred video therapy did not know that Talkspace provides video therapy.
	Relational	A South Asian male participant stated, “where I come from, you would never talk about mental health.”
**Action planning**		
	What platform?	Participant had also tried Noom and BetterUp, at times concurrently.
	What treatment?	Participant who was self-conscious about her appearance preferred messaging only.
	What provider?	Participant would like to see more demographic variety in available therapists.
Treatment		Participant was happy that she was able to message her therapist whenever she felt strongly about something.
**Therapeutic alliance**		
	Real bond	Participant primarily used the platform to gain a sense of connection (when she had been drinking).
	Goal agreement	Participant and therapist agreed early on in treatment to aim toward being less anxious at work.
	Process agreement	Participant liked that her therapist would respond in the same format that she responded to him in (bullets).
Therapeutic rupture		Participant felt that the second was too generic and repetitive with advice that did not work—somewhat like a bot.
**Rupture responses and coping planning**		
	Replace provider	Participant was demoralized when they had to switch providers and retread old conversational ground.
	Repair problem	Participant did 1 video session, did not like it, and switched to messaging only with the same provider.
	Withdraw	Participant felt as though she was not being listened to and stopped responding as frequently.
**Termination**		
	Successful	Participant used Talkspace for approximately 4 months and ultimately felt that she got what she needed and did not need to renew her subscription.
	Unsuccessful	Participant stopped treatment because she did not think her provider had an authentic understanding of her particular life conditions.

### IE Model

Here, we provide a brief overview of the IE Model, followed by a detailed summary of each construct with qualitative evidence. This new model consists of 4 phases: deciding, selecting, engaging, and terminating. Per the IE Model, potential users start out in a (1) *deciding phase* wherein they first form a behavioral intention to engage in some form of DMH treatment. An individual’s behavioral intention formation is influenced by both their negative and positive outcome expectations related to treatment as well as 3 principal appraisal-motivational factors: the individual’s perceived level of need for mental health care, the individual’s positive or negative attitudes toward care, and the individual’s domain-specific self-efficacy related to the tasks that involve DMH treatment. These intrapsychic factors are influenced by external resources and barriers that are segmented into 5 categories: financial, geographic, informational, technical, and relational. Once an individual forms a behavioral intention to engage in treatment, they enter the (2) *selecting phase*, wherein they attempt to answer 3 core questions: “What digital platform should I use?” “What treatment plan should I use?” “What provider should I work with?” Each of these questions is influenced by appraisal-motivational, outcome expectancy, and resource and barrier elements from the deciding phase. These questions, once answered, lead to the (3) *engaging phase*, which begins with treatment initiation and transitions to treatment maintenance. Treatment initiation and maintenance are influenced by external resources and barriers, appraisal-motivational factors, and therapeutic alliance. Over the course of treatment, it is possible that a therapeutic rupture—a breakdown in the therapeutic alliance—takes place. At this point, the user has 3 possible rupture responses. They can replace their provider, thereby returning to the selecting phase, they can attempt to repair their relationship and reinitiate effective treatment, or they can withdraw from treatment, leading either to the unsuccessful termination of treatment or an eventual reinitiation of treatment after a period of dormancy. Even if someone continues in treatment with a provider, they will eventually reach the (4) *termination phase*, with termination existing on a continuum from successful to unsuccessful. The outcome of treatment will inform an individual’s future outcome expectancies regarding mental health treatment, as well as their appraisal-motivational factors.

### Appraisal-Motivational Factors

#### Overview

A set of intrapsychic, appraisal, and motivation factors (ie, “appraisal-motivational factors”) played a considerable role in participants’ digital help-seeking and treatment journeys. These were as follows:

- Perceived needs: an individual’s perception of their level of need for engaging in digital therapy
- Treatment attitudes: cognitive appraisals or a way of thinking or feeling about digital therapy. This component was an addition to the HAPA model.
- Self-efficacy: perceived behavioral control or an individual’s beliefs about their ability to perform the tasks necessary to engage in digital therapy

#### Perceived Needs

When asked about why they sought digital treatment, all participants noted a perceived mental health need. In some cases, this was a function of prior experiences with mental health emergencies:

I’ve had three suicide attempts in my life and one ICU event, so I completely understand at this point in my life that I need help–that I have to talk to someone because the way I think of things and process it on my own can lead to bad end results.

Other participants noted more general and less immediately urgent life difficulties, and they believed themselves to be unable to manage their mental health challenges effectively by themselves:

And I was just in at the time, I was really struggling and really just needed somebody. And I thought, you know what, I’m just gonna try it, we’ll see what happens.

#### Treatment Attitudes

Participants held a range of preexisting attitudes toward DMH. Some harbored no stigma regarding mental health treatment, resulting in positive attitudes toward their sessions (eg, “I look forward to connecting with my therapist every day.”). These attitudes varied over time, becoming more negative upon unsatisfying interactions with their provider and more positive when users felt listened to, cared for, and connected to their therapist. One user particularly attributed positive sentiment toward treatment to her ability to engage continuously with her provider via text:

And so [...], you know, I could, anytime, write out what I was feeling [...] that kind of just adds a nice dimension to sharing your experience with the therapist.

Some participants noted that internalized mental health stigma had made them reluctant to seek help. One participant with a long history of mental health treatment and high perceived need disclosed generally skeptical attitudes toward the authenticity of the emotional intimacy she may have experienced at different points during her DMH treatment experience:

Like I said, I’ve always thought of talk therapy as being almost like, please forgive me for using this word, but like, talk prostitution, like I pay you and you let me talk to you.

That this participant used Talkspace despite her negative treatment attitude indicates that, at least in some cases, mental health help seeking can coexist with negative treatment attitudes, especially given high perceived needs.

#### Self-efficacy

Participants’ reluctance to try the digital platform was partly due to their lack of confidence in their ability to effectively engage in the treatment. Low self-efficacy in *receiving* digital care–informed participant treatment. For instance, 1 participant, doubting whether she would be able to remain present during video sessions due to her self-consciousness, opted for a purely text-based treatment. Another participant indicated low confidence in their ability to accurately express themselves in real time and accordingly chose a messaging-only treatment plan:

...being able to write it out was much easier for me to convey what I wanted to communicate to my therapist.

Although self-efficacy played a role in DMH engagement, focus group data did not enable us to distinguish between the 3 subtypes of self-efficacy outlined in the original HAPA model:

- Task self-efficacy: beliefs about one’s ability to engage in health behavior activities.
- Maintenance self-efficacy: beliefs about one’s ability to cope with barriers that arise during a health behavior maintenance period.
- Recovery self-efficacy: beliefs about one’s ability to resume health behavior after a lapse.

Participants’ self-efficacy narratives centered on their ability to use the app itself and build a therapeutic relationship with their therapist. There was some debate among coders as to whether 2 different subcategories of self-efficacy should be defined: “technical self-efficacy” and “relational self-efficacy.” Ultimately, the coding team decided to define only 1 overarching construct of self-efficacy because of the paucity of participant references to self-efficacy.

### Outcome Expectancies

#### Overview

Outcome expectancies are people’s expectations regarding the clinical outcomes resulting from engaging in DMH treatment. These differ from attitudes because they are beliefs and focused on treatment outcomes. Participants described both the potential benefits of treatments, such as interpersonal connection and symptom reduction, as well as the drawbacks of treatment, such as wasted time, financial costs, and the fear of being neglected by their therapist.

#### Treatment Positives

Those who endorsed higher initial outcome expectancies were more likely to have positive prior experiences with therapy and to focus on the ways in which the experience of digital therapy could be similar to that of in-person therapy:

I figured, well, Talkspace would just be, you know, generally the same thing if I got a traditional therapist versus something, you know, on this on this medium, so I was just as confident as I would be in any other kind of therapy.

Other participants recalled being excited by the prospect of engaging regularly with a provider from their mobile phones and were optimistic that on-demand connection with a mental health expert would be helpful.

#### Treatment Negatives

Those who reported more mixed and negative outcome expectancies were focused on the potential differences between the experience of digital versus in-person treatment. Participants worried that the lack of verbal cues and body language in messaging therapy could interfere with the patient-therapist relationship:

So, I’ve done therapy in person before trying Talkspace. And so I was really concerned if, if I would really be heard the same way – if I would have like the same connection, if that would all be there. And you know, if like, body language [...] and all of that would really be able to read and understood.

### Resources and Barriers

#### Overview

Participants identified several types of external resources that facilitated their engagement in digital treatment, as well as barriers that led them to either disengage or fail to initiate treatment. This finding maps well onto work in the human-computer interaction field related to the burden associated with using technology. According to Suh et al [50], user burden consists of 6 constructs: challenges in understanding how to use the tool, physical challenges in using the tool, time taken to engage with the tool, mental and emotional burdens, concerns with privacy, and financial burden. On the basis of our focus group data, the burdens identified by participants when using DMH are financial, geographic (which may align with physical and time burden), informational and technical (which align with user understanding), and relational (which align with mental and emotional burdens). We propose segmenting a preexisting feature of the HAPA framework—the construct of “external resources and barriers”—into these 5 subconstructs.

#### Financial

Many focus group participants noted that the financial cost of DMH treatment was a barrier and one that led many to terminate their treatment despite psychological benefits. Conversely, participants with workplace-related benefits packages, including a partial or fully paid subscription to the platform, were more likely to continue to engage in treatment. One participant’s experience exemplifies the benefits of workplace-assisted mental health packages on initial treatment engagement:

It’s one of those things where it’s like, my employer is offering it, having someone that actually has experience with it. It’s like, okay, this is fantastic. I’m gonna go ahead and give it a try. Awesome.

The presence or absence of financial resources played an important role in digital engagement. Focus group participants who had access to substantial coupon codes that could be redeemed only during the first month of treatment described ending their subscription once these coupons ran out, even when they found the treatment to be very helpful.

#### Geographic

In this focus group, participants identified the ability to use the platform from anywhere as a means of overcoming geographic barriers to treatment. Several participants located in more remote areas of the United States noted that in-person therapy presented a time burden that exacerbated last-minute cancellations:

You don’t want to have to drive an hour to therapist and then find a little sign on our door saying that, you know that sorry, you didn’t get my email, but I’m not going to be here, you know, or something like that.

When asked about her decision to seek therapy on a digital platform, another remotely located focus group participant noted that DMH treatment was her only viable choice:

And I thought “I just have to talk, I have to talk to someone.” Like I said, I live out in the back of beyond. I’m the last driveway for 60 miles. So that is what my thought process was in answer to your question. It was just, I got to talk to somebody.

### Technical

#### Overview

A few participants noted technical challenges due to the platform itself (eg, a mobile app bug) or their resources for connecting to it (eg, sparse internet connection). For 1 geographically remote client, internet-related technical difficulties posed a continuous barrier: “It’s just one of those things that I will say is hard because you just can never guarantee what your internet service is going to do.” Most participants encountered minimal technical difficulties and agreed that the in-app user experience was intuitive and simple to navigate.

#### Informational

Informational barriers and resources touched on Talkspace’s platform and individuals’ knowledge of mental health and mental health services. Access to information about Talkspace’s menu of treatment options was a resource that some users lacked; 1 focus group participant suggested that Talkspace expand its services to include live video sessions and was surprised to learn that the platform had been offering video-based therapy throughout her time on the platform.

#### Relational

Several participants noted that their DMH engagement was either positively or negatively affected by personal relationships. One South Asian focus group participant, wanting to keep his mental health issue from his extended family, felt a digital platform would be his most discrete treatment option: “...where we come from, you know, we don’t—we can’t even tell the family that we’re going to therapy.” Relationships were also a resource and facilitator of treatment. Another user reported that he would often speak with his partner immediately after the end of his weekly therapy session to share his newfound personal insights.

### Behavioral Intention

According to the HAPA model, engaging in health-promoting behaviors involves a motivational stage, where an intention is formed, and a volitional stage, where planning and execution take place, broadly corresponding to the deciding stage of the LIM. After a behavioral intention is formed, in the volitional phase, the individual begins planning the specific actions needed to facilitate change. In the context of DMH treatment seeking and engagement, these 2 phases are chronologically truncated into 1 continuous process, that is, users with basic resources (eg, a device with access to the internet) can form an intention to seek DMH treatment and then immediately take the next steps toward entering treatment. Accordingly, it proved difficult for coders to differentiate between instances of behavioral intention formation and instances of action planning. When asked about how they initially formed a decision to seek help, individuals had a variety of responses. Some focus group participants reported coming gradually to an intuitive understanding that seeking *some* kind of mental health treatment was needed: “I decided [Talkspace] was something I needed...It just felt like it made a lot of sense for me, especially with my work schedule.” Other times, behavioral intention formation was triggered quickly in response to seeing an external cue, such as an advertisement for the platform over social media:

I saw an ad and was kind of like “okay, I should do this now.” And I think that was probably the easiest part...just being able to kind of start the process immediately without having to look online and get referrals for someone locally.

This participant’s example also highlights how when the behavioral intention is inspired by learning about a particular tool, such as through word of mouth or an advertisement, the selection and deciding phases of the LIM may be concurrent, or selection may even precede the decision to take action. In addition, as we have discussed further, the process of investigating Talkspace or another DMH tool to support a decision sometimes includes taking initial steps toward action planning or initiation, such as creating an account on the website.

### Action Planning

#### Overview

Nearly all focus group users reported that they began planning their DMH treatment journeys immediately after forming a behavioral intention to seek treatment. Once users decided that they should engage in DMH treatment, they immediately began an internet search to ask and answer questions about the type of platform, treatment, and provider they should use.

#### What Platform Should I Use?

Participants generally began their action planning by asking themselves which DMH platform was best suited to their needs. Users visited websites, read reviews, and downloaded multiple apps.

Participants noted being attracted to the prospect of being able to engage in treatment at their own pace and saw Talkspace as a platform where the user could architect their DMH journey:

...it was customizable...[Talkspace] was accessible, I could send as many or as little messages as I want. I could follow up when I needed to or when I wanted to.

Because our sample is restricted to individuals who chose to use Talkspace, we were unable to derive firm conclusions regarding the factors that are most important for *all* potential users in determining which platform fits their needs, but our focus groups indicated that users were interested in privacy, accessibility, and credibility.

#### What Treatment Package Should I Choose?

On Talkspace, DMH treatment seekers also decide which treatment package they should try, generally divided into text-only, text plus once-per-month video sessions, or text plus once-per-week video sessions. Our participants noted a range of preferences for different treatment options on the platform. One participant’s choice of messaging-only treatment plan was influenced by self-consciousness about her own appearance*:*

...it all turned out to be text or audio, which I think I preferred. I have a tendency to be really self-conscious.

Several other participants indicated that asynchronous messaging fits well with their schedule and offered a uniquely “on-demand” therapy experience:

I think the chat feature was what made Talkspace unique. It was what I really liked about it...I just really think that that chat feature is unique and, you know, you know, not something that you really can replicate from, you know, [offline] therapy relationships.

#### What Provider Should I See?

Choosing the right provider was one of the most important decisions that most participants felt they had made throughout their treatment journey. Participants noted that Talkspace offered a wide selection of potential providers. Most participants had positive things to say regarding their provider selection experience, such as 1 person who noted, “I liked that I could go through the available counselors, and then can choose the counselor which seems best for me.” Another participant reflected that her therapist selection decision was decided by a sense of connection rather than credentials:

I liked how y’all have the picture [of the therapist]. And then the specialty of what they do, and then they write about themselves. And so when I was reading that the reason I chose [my therapist] is because of her wording. It is the way she wrote her introduction that got me. And I liked the way she put it out there. Some therapists were just like, “I've got a degree. I’ve done this.” And I don’t care about that. The way she wrote it was inviting. And I just I felt like that’s going to be a good match. And it did it ended up being perfect.

Some participants expressed the desire to match with a provider with similar racial or ethnic backgrounds:

For me, I think the biggest one was finding a therapist who would understand my unique experiences and backgrounds. So just having a diverse, diverse selection of therapists, I remember when I first like was going through therapist bios to see their background, I was definitely looking for an Asian woman as a therapist. So that I think that was one of the concerns I had going into it.

### Treatment

#### Overview

After matching with a provider, participants entered treatment and the engaging phase or what the LIM would describe as “tracking and acting.” Drawing from the original HAPA framework, treatment can be segmented into 2 stages: initiation and maintenance.

#### Initiation

Whereas the initiation of traditional psychotherapy will almost always entail a discrete event (namely, an initial therapy consultation or face-to-face session), the boundary separating DMH treatment initiation from prior action planning steps is not so clear-cut. This is because users so often initiated treatment very shortly after they formed a behavioral intention. We operationalized treatment initiation as the immediate steps that take place after a user signs up for a specific treatment package on the platform.

Although the ability to rapidly move from behavioral intention formation to treatment initiation is a clear benefit of DMH platforms, 1 participant noted his ambivalence regarding the speed of this process:

I found myself [signed up for the platform] within a span of like, 10 minutes [...] You know, I already had scheduled something, I mean, it was just, it was actually almost too simple. But I also think that made me feel more comfortable about it, because it was so easy to do. [...] I didn’t feel like I had to jump through hoops or, you know, cut red tape or any of that stuff.

Streamlining the transition from intention formation to treatment initiation may have drawbacks in addition to its obvious benefits. Users might benefit from more reflective deliberation before selecting a platform, treatment modality, or provider to ensure that their choices align with their treatment goals and preferences. There may be an inevitable trade-off that platforms need to consider between providing critical treatment information and focusing on treatment initiation.

#### Maintenance

After treatment initiation, participants were free to engage with providers. Unlike in traditional psychotherapy delivery settings, this engagement could happen at any time that the DMH user finds convenient. One participant was happy that she was able to message her therapist whenever she felt the need:

I know, for me, the thing I really liked about Talkspace was the fact that I could do the text messages like anytime to my therapist, like anytime anything came up. So, I could just immediately go send that to them texts and like notify them. And I also did do some chatting, but not video chatting, but I would get overwhelmed with talking. So being able to write out my thoughts made it much easier for me to convey what I wanted to communicate to my therapist.

Although participants were universally in favor of this constant ability to message their provider on demand, many of the same participants reported a period after downloading the app when they realized how much the experience of digital therapy differed from in-person therapy. Participants who expected on-demand *responses* from their therapist were more likely to report having unmet treatment expectations, particularly in the earlier stage of the treatment. Another subset of participants with high outcome expectancy, generally with prior experience in therapy, had no preconceptions regarding the nature of the digital therapeutic experience. This latter group of participants tended to report being pleasantly surprised by how well they were able to “customize” their treatment experience to suit their therapeutic needs:

So, I was initially thinking “Okay, [let me] kind of like try this out. Let’s see how it goes.” And it ended up being so impactful. For me, it worked for me, I could see how it wouldn’t necessarily work for everyone. But it was customizable. It was accessible, I could send as many or as little messages as I want. I could follow up when I needed to or when I wanted to. So, it was a really good thing for me.

### Therapeutic Alliance

#### Overview

One of the most consistent discussion topics throughout the focus groups was participants’ relationships with their provider. Although the focus group sessions were not intended to explore therapist-participant dynamics, these dynamics were central determinants of the participant’s DMH engagement. Accordingly, the 3 components of the therapeutic alliance (ie, goal agreement, process agreement, and the development of a real bond) were analyzed.

#### Real Relationship

Many participants came into treatment focused on developing a genuine relationship with their therapist, and therapists’ initial messages seemed to have a large impact on users’ beliefs regarding the feasibility of developing a bond over text. Some users reported instantly “clicking” with their provider, while others felt ambivalent about their therapist’s responses, and still others were immediately disappointed. Short and generic-sounding therapist responses caused participants to quickly lower their expectations. As another user put it, “I kept getting the same cookie-cutter, candy-canned messages!” Yet other users were surprised by their therapists’ thoughtfulness. One participant who was struggling with substance use noted the following:

I didn’t know what to expect honestly, but I didn’t expect how thorough [my therapist] was going to be on the front end of the first session. Understanding, you know, everything that I wrote and asking specific, pointed questions about things that I wrote.

Although users generally agreed that the message-based communication allowed them to develop genuine relationships, several users felt that they could have developed a stronger relationship with their provider if they had engaged in an introductory video session. Conversely, some users who *did* engage in these introductory video sessions felt that the visual and synchronous components did not materially add to their experience. One user said, “I did a face video once with [my therapist]...she’s precious, but I don’t need to look at her.”

#### Goal Agreement

Although therapist-client goal agreement was central to participant experiences, participants’ therapeutic goals were often not made explicit. One participant who had clearly defined treatment goals found her therapist to be helpful early on in her DMH journey:

My goals at the time were to kind of deal with anxieties that were cropping up around the pandemic and how I was dealing with all of that, and [my therapist] really proved to me that she was listening by how she responded with detail and with care.

Conversely, 1 participant voiced frustration that his treatment goals were not explicitly discussed:

For me, my experience with my therapist was like, “How are you feeling today?” And like, that’s a really good opening question. But then there was nothing about “What are your goals for this session?” “What are your goals for this month, and maybe even something to help build towards?” You know, “What do you want to work on this month?” Or “How are you feeling through this?,”...something like that could build some type of achievable outcome.

Notably, several participants recommended that the platform should include the ability to have clients enter therapeutic goals in their profiles that their therapists could regularly reference. These accounts suggest that goal agreement is an important early feature of an effective healing relationship and that therapeutic dyads that more explicitly outline and return to a client’s goals will engender a sense of progress and purpose, thereby leading to continued engagement.

#### Process Agreement

Although participants expressed confidence about the type of therapeutic responses they thought would be most helpful, these thoughts differed; some preferred a nondirective approach, whereas others explicitly sought advice. When their providers aligned with their preferred treatment style, participants reported feeling more optimistic and engaged. One participant seeking help with depression was happy to receive recommendations from her provider:

So it was really nice to kind of get some of that advice, and kind of put that into practice and then have an opportunity to also connect.

Another noted that “helpful advice was something that I thought was very beneficial.” However, other participants wished that their therapists gave less advice and felt that the advice, when given, was overly generic. One participant who described herself as “desperate for connection” grew particularly frustrated when her therapist offered suggestions about how she could cope with her challenges. It follows that, by explicitly asking about these preferences, DMH providers can tailor their directiveness to suit the needs and preferences of users.

Implicit agreement on the therapeutic process was also reached in terms of messaging styles and cadence with which the therapeutic dyads engaged. Dyads who explicitly outlined communication expectations (eg, “I will respond to you at least five times per day, five days per week”) were more likely to result in positive treatment experiences. The messaging style was also important:

So, I could kind of talk about a few different things. In the context of me sending one message, you know kind of just bullet format it out, and then get responses instead of just responding to one of those things. [My therapist] could respond individually back to me to each of those topics.

### Therapeutic Rupture and Responses

#### Overview

The notion of therapeutic rupture and repair has a long history in face-to-face psychotherapy process–outcome research, but its impact on engagement in DMH treatment has not yet been thoroughly explored. Many participants described the periods in which they felt disconnected from their provider. These periods most often centered on the content or style of therapist messages. For instance, 1 user noted as follows:

[My therapist] was just giving me generic information. And there were a couple times I was bringing issues to her and she would repeat something she’s already told me that I told her did not work prior. So, it’s just like, “Oh, she’s not really listening to my concerns,” like “she’s working from a script.” So, I was worried that she wasn’t really listening to what I’m saying. She’s just like seeing keywords...it was like a bot.

Overly generic, short, cookie-cutter messages, such as those mentioned earlier, tended to prompt these therapeutic rupture moments. Focus group participants responded to these moments in three primary ways: (1) seek to replace their current provider; (2) address the rupture to repair the relationship; or (3) withdraw from treatment, either actively or passively.

#### Replace the Provider

Some participants opted to replace their providers when they felt a therapeutic disconnect, whereas others did not seem to mind having to switch providers or even to do so multiple times. One participant casually reported:

I also ended up switching and went through two therapists before finding my third one, who I love.

Another was appreciative that the platform made it easy to switch providers:

I think after it was like one or two sessions, I was like, this person just doesn’t get me. It’s nothing personal. They just have their own opinion of it. And I enjoyed being able to ask, you know, to kind of send a message and easily say like, this isn’t the right fit, because, and then they helped match me with someone else who worked really well with me for a while.

The ability to instantaneously terminate with one provider while engaging with a new one is both a unique feature of DMH platforms and a well-appreciated aspect of this user experience:

My ability to change to a different therapist was phenomenal. Like, there was no questions asked, I mean, it was just kind of like a little survey. And the survey was very quick. It wasn't, I didn’t have to write a dissertation to explain why I was just able to change. And, and that helped me tremendously.

However, for the other participants, switching providers was a thoroughly demoralizing experience. Several participants said that the switch required them to “retread old conversational ground.” Demoralization was highest in cases in which participants had already invested time and energy in working with their initially assigned provider. Some active users might persist with a provider even in the absence of a strong therapeutic alliance because of the time and effort it takes to make a therapist switch. However, participants who switched providers often found better dyadic experiences after switching.

#### Repair the Problem

Although many focus group participants opted to replace their provider, fewer seemed willing to directly address the source of their therapeutic rupture. Participants who addressed ruptures were centered mainly on aspects of the platform experience (eg, switching from messaging-only therapy to a video-based plan) rather than aspects of the therapist-client relationship. Although participants found it easy to request a change in their platform experience, such as using text instead of video, they appeared less willing to ask for a change in the provider’s therapeutic approach (eg, more messages per day and less advice). At times, the line between rupture repair and provider replacement was not clear. One participant initially confronted his provider about her apparent lack of thoughtfulness, tried to replace her as a provider, and left the platform:

I cancelled Talkspace for about a month, and I dismissed...my therapist. She, she gave me one-line responses to some of the problems I had, and she proposed some very frustrating psychobabble, which is, you know, it’s not really psychobabble. But it’s just basically anyone already knows...And I, I confronted her on that. And then that that was it. That was a Thursday, she didn’t reply to what I had said for the rest of Friday, and I just threw my hands up. And, I switched to a different therapist at that point in time.

Interestingly, this user left the platform, returned, and ultimately rematched with the original provider, reporting the following:

Our relationship has improved over time over many months. And she and I have a lot of similarities in the way we think, and our personalities and our perceptions...She now gives me very, very well thought out research and analysis and she understands more.

#### Withdrawal

Many participants opted to withdraw from treatment rather than address their concerns directly with their provider or seek another therapist. This withdrawal was generally passive; participants reported simply disengaging from the platform. One participant, unhappy with the directive approach her provider was taking, eventually disengaged altogether:

I think ultimately, I stopped using Talkspace because they were like, really heavily pushing CBT methods and like, those just weren’t working for me. So maybe if like, there was something else, like the therapists were trained in, or if there was an option to like, just talk and like, you can stop pushing the like, reframing and everything. Like that approach doesn’t work for everybody, you know?

Passive withdrawal is another approach:

I used it for about a month, but then I just let my three-month period run out. So, I didn’t actively go in to cancel it. I had maybe thought about switching therapists but it felt like doing the switch and only having six to eight weeks or so to like start a new relationship again, didn’t feel like enough time to make it worthwhile.

Withdrawal can occur even when a user has a positive bond with their therapist. One participant who had been struggling with a low sense of self-worth stated the following:

My therapist was lovely. She was just very upfront about how [I should be] typing about [my] experiences...one of the things I really needed help with at the time was like taking space, I found that it was really hard for me to interact with my therapist just because I was like, “Well, what if she’s busy?,”...even though there was all this freedom [to message] and that was a great thing.

Withdrawal and replacement may be more common responses to therapeutic ruptures in DMH than engaging in relationship repair. Notably, rupture-repair experiences have been shown to be associated with better clinical outcomes in face-to-face psychotherapy [43]; this prior literature suggests that the relative ease with which a user might disengage from a ruptured relationship in DMH contexts could ultimately be to the detriment of the therapeutic experience. This would also align with the research on engagement with digital tools that informed the LIM. As noted by participants in focus groups, there are often fewer barriers to initiating the use of a DMH tool or treatment. Although we note the opportunity to scaffold slower, more intentional choices of DMH treatments, therapists, and tools, it can also be advantageous to see as one of the benefits of DMH: people can try a tool, treatment, or therapist to determine if it works for them. If it does not, they can switch. To achieve these advantages, we also propose design opportunities informed by the LIM: designs that support people in assessing whether a tool, treatment, or therapist is working for them (while also not promoting unrealistic expectations about immediate successes), and techniques that support tuning engagement to make an experience work better or return to the selection and initiation stage if someone should try a different tool, platform, or therapist. In addition, when people learn that a particular approach is not working for them, designs should frame this disengagement and reengagement as a success—they have learned something—not a failure.

### Termination

Although many health behaviors (eg, quitting smoking and exercising regularly) are lifelong endeavors, effective psychotherapy usually reaches an end point. The focus group participants described an array of factors that led to their terminating therapy. Therapy termination can be conceptualized on a continuum from “successful” to “unsuccessful.” Successful termination is characterized by the participants’ sense that they had made considerable improvement while using the platform and that this improvement was connected to their treatment. One client used the platform for several months before ultimately deciding that she had obtained what she needed from the intervention:

Yeah, I think I used the platform probably between three to six months. I think I had like kind of like an extended subscription. And you know, when it came time to renew, I kind of felt like I had gotten what needed out of it at the time.

Consistent with research informed by the LIM [39], DMH tools should recognize these successes. This extends to reminding someone of their past success if they later reengage in treatment.

However, clinical improvement was only one of the many reasons why participants terminated. Other participants indicated that while they generally had positive experiences with the platform, they eventually realized that they needed a different treatment approach. One participant left the platform to take a more explicitly spiritual approach to treatment:

I was kind of apprehensive to the idea of virtual counseling. But Talkspace actually ended up being a really good option for me...the only reason that I did stop working with Talkspace is because I was I kind of just got to a point where I felt like I, I needed some faith-based counseling, that, you know, just something that aligned more with my beliefs...the [therapist] that I was talking to was really helpful. But as we’re talking, I started realizing that they were just things on a deeper level that I needed, like spiritually.

Most participants cited financial cost as a key factor that impacted their decision to continue on the platform. As indicated in previous participant quotes, several others felt driven to end therapy because of the perceived lack of helpfulness of either their provider or the broader platform experience. Critically, the decision to terminate treatment, even in the absence of substantial improvement, may impact an individual’s beliefs about themselves and treatment (ie, their appraisal-motivational factors) and, in turn, their likelihood of reengaging in any treatment. This is illustrated in the IE Model, with arrows from termination to appraisal-motivational factors and outcome expectancy. One participant exemplifies how unsuccessful treatment can lead to lowered self-efficacy in the domain of DMH treatment engagement:

I felt like maybe I’m not able to really communicate well through this platform. And I mean, it truly didn’t feel like a competence issue on my therapist part at all. I just felt like I wasn’t really doing well with the texting.

## Discussion

### Principal Findings

At present, there is no widely agreed-upon model of engagement in digital psychotherapy or an explanation of the mechanisms by which engagement affects clinical outcomes in DMH. In this study, we synthesized the HAPA and LIM frameworks, along with interpersonal constructs from face-to-face psychotherapy process–outcome research, to offer an IE Model of digitally delivered psychotherapy. Although our findings emerged from user focus groups on a single digital psychotherapy platform, we believe that this model is transferable to other clinician-guided DMH settings and provides a useful conceptualization of engagement and disengagement in guided DMH interventions.

Although our focus group findings were generally consistent with the HAPA model and LIM, we made several changes to these frameworks to account for distinct aspects of the digital therapy user experience. First, we eliminated the construct of risk perception. In the original HAPA model, risk perception includes concerns about negative consequences from *not* engaging in a health action. In our focus groups, no participants indicated that they considered the risks of not seeking treatment as an active element in their decision-making. Hypothetically, fears about deteriorating personal relationships, impacts on employment, or increased suicidal ideation from foregoing mental health treatment could have been coded as instances of risk perception had they been voiced by any of the focus group participants. The notable absence of these comments is consistent with a quantitative meta-analysis finding that risk perceptions have small or no effects on behavioral intention formation and health behavior enactments, which has led to a proposed truncated HAPA model that does not include risk perception [30].

Second, we found it necessary to incorporate interpersonal constructs, such as the therapeutic alliance, to adequately model the relational drivers of digital therapy engagement. Almost every participant in the focus group commented that their ability to connect with their therapist was a deciding factor in their treatment. Accordingly, in the IE Model, the platform itself was reconceptualized from a treatment in its own right to a *treatment conduit* that facilitated access to a *helping individual*. In other words, users did not see themselves as engaging with a *platform* as much as in a healing relationship. The platform generally functioned the best by removing barriers to access and expanding the selection of providers and treatment options. Concerns over treatment were often related to therapist factors (eg, lack of responsiveness and lack of relevant expertise) or relational factors (eg, doubt over fostering an authentic relationship over digital media). When designing or evaluating digital tools, it can be tempting to focus on the tool design, but the interpersonal context of their use remains important for their success or failure and continues to require attention from researchers and designers [49]. Any model of help seeking and health behavior in messaging therapy must consider the interpersonal factors inherent to the provision of any psychotherapy intervention to sufficiently account for the drivers of user engagement or disengagement. This can inform design strategies for supporting the interpersonal relationship as well as for assessing when a client-therapist relationship is not working and shifting to a different therapist on the same platform.

Third, we incorporated the LIM, which added considerations of termination, cyclical engagement, and iterative impact of treatment experiences on precursors to treatment decision-making. Unlike many other health promotion behaviors (eg, exercising and eating healthy foods), engaging in psychotherapy is not a lifelong endeavor. Indeed, one mark of successful psychotherapy is its timely completion. Although a systematic account of the determinants of successful therapy termination is beyond the scope of this study, 2 insights come about from our focus groups. First, the decision to terminate is informed by a variety of intrapsychic, relational, and external (eg, financial) factors. Second, each digital therapy termination can be usefully framed on a continuum from wholly unsuccessful to wholly successful. Critically, digital therapy offers an ease of termination via withdrawal and reengagement and cycling via changing providers, which is not available in traditional therapy. This feature may facilitate the identification of high-quality matches and thereby lead to better outcomes, but it may also decrease client and therapists’ efforts to repair ruptured therapeutic relationships. This reduction in relationship repair efforts by either the therapist or client may be a step backward, as relationship repair has been a central therapeutic process in face-to-face psychotherapy [51].

The IE Model offers a large step forward in conceptualizing predictive, causal explanations for engagement and disengagement in services and offers practical implications for mental health service providers and platforms. Our results highlight how the range of ways in which people might engage with even a single DMH feature (eg, variation in messaging frequency, tempo, and length), combined with the delayed feedback that can be present in asynchronous interactions, increases the importance of designing supports for process agreement. Service providers seeking to engage clients can use messaging and design options that activate appraisal-motivational and outcome expectancy factors. These might include developing web-based screening tools to help clients identify their needs, streamlining the treatment process to increase self-efficacy, and using a destigmatizing language to improve treatment attitudes. Clients can be offered a chance to discuss any poor past experiences they have had with treatment to address any concerns and negative expectancies. Notably, these recommendations around eliciting user engagement are agnostic to any particular digitally delivered psychotherapeutic modality (eg, internet-delivered Cognitive Behavioral Therapy, digitally delivered Person-Centered Therapy, or internet-based psychodynamic psychotherapy). The IE Model can apply to every form of digital psychotherapy because every form of digital psychotherapy involves interactions between the patient and provider as well as a set of agreed-upon therapeutic tasks in which the patient should engage.

Participants in our study described how the immediacy of starting treatment in a DMH could be beneficial but how they might have also benefited from more reflective deliberation before selecting a platform, treatment, or therapist, and here, we highlight the concept of *design friction—*microboundaries that create opportunities for reflection [43,52]—and how embedding such features in DMH platforms could lead to better alignment with a client’s goals and needs. However, adding barriers comes with the risk that fewer people will initiate treatment, and so further research is needed to assess when and what kinds of frictions are beneficial.

Moreover, as highlighted by the IE Model, resources and barriers can be carefully considered before and during treatment to ease treatment initiation and support maintenance. Moreover, DMH platforms can offer design solutions for building the therapeutic alliance by dashboarding clients’ goals—the jointly agreed-upon tasks that therapists and clients engage in to address those goals—and clients’ outcomes. Reminder systems can automatically steer attention back to the dashboard, so lack of progress or potential therapeutic ruptures can be addressed in treatment before premature disengagement occurs or so a new therapeutic match can be formed to support the client more appropriately. The model can serve as a guidepost for service providers to optimize engagement in care.

Similarly, researchers studying engagement in digital psychotherapy can now refer to a comprehensive model to articulate the barriers, facilitators, and general determinants of treatment engagement. Many digital treatments that might otherwise prove efficacious are hindered by insufficient treatment engagement. This model provides the factors and underlying processes that explain digitally guided treatment engagement, describing many putative mechanisms driving user behavior on these platforms. This may be particularly important to understand in naturalistic DMH outcome studies, where data are often collected passively and remotely. In these contexts, the drivers of engagement in DMH research (eg, filling out self-report batteries) may largely overlap with the drivers of treatment engagement.

### Limitations

This study had several limitations. First, the focus group size (N=24) was too small to serve as an exhaustive sample for the variety of user experiences on digital therapy platforms. Our focus group participants described a highly diverse range of experiences and engagement trajectories, and it is possible that a larger sample would have uncovered more constructs or otherwise led researchers to alter the IE Model. The focus group sample was also nonrandom and, similar to any study using actively collected data, was likely impacted by selection bias toward people who are willing to engage in focus groups. Furthermore, our reliance on focus group data precludes us from considering factors that individuals either do not have conscious awareness of the focus group setting (eg, latent negative attitudes toward treatment) or do not feel comfortable disclosing in the focus group setting (eg, a preference for a provider with a similar ethnic background). These limitations may have led researchers to oversimplify some facets of the proposed IE Model. For instance, it is not clear whether self-efficacy in the context of digital therapy treatment seeking and engagement can be usefully segmented into *technical self-efficacy* and *relational self-efficacy*, and it is possible that further research will confirm that there is a useful conceptual difference in this case. Similarly, there remain open questions regarding the boundaries demarcating both IE Model phases and IE Model constructs, which we leave for future work. Perhaps most critically, these preliminary findings are derived from focus groups of users on a specific 2-way asynchronous digital messaging platform; any extension of the IE Model to other DMH contexts should be undertaken cautiously and on an exploratory basis. Therefore, it is unknown whether this model applies to other types of DMH interventions, such as mental health chatbots (eg, Woebot or Wysa) or other digital psychotherapy platforms. Finally, our sample was drawn primarily from users who have not had prior experience with any form of psychotherapy. It is likely that prior experiences in mental health treatment, whether digitally mediated or in-person, would greatly impact users’ expectations regarding therapeutic digital platforms, and any such expectations could not have been adequately captured, given our present sample.

### Future Directions

With the IE Model as a theoretical backdrop, future research on the question of engagement in digital therapy falls into at least 3 categories. First, it is necessary to test the proposed IE Model using quantitative longitudinal data collected from users’ platform experiences. Given the many interacting constructs proposed in this model, dynamic structural equation modeling (DSEM) appears to be an especially fruitful and fitting data analytic procedure. DSEM has several potential advantages, including the ability to model complex, time-dependent relationships between variables and the capacity to estimate within-individual and between-individual variation simultaneously [53]. DSEM may be particularly useful in identifying patterns of optimal engagement that lead to particularly successful clinical outcomes for subsets of users. Although this method requires large sample sizes and observations at multiple time points, such samples are relatively more readily attainable in the context of low-to-no-cost digital interventions, as compared with in-person mental health treatment contexts.

Second, there may be an implicit assumption in some corners of the intervention science field that DMH engagement patterns should be similar to those observed in traditional mental health treatment contexts. However, we view this assumption as problematic. If we are to be effective in developing new digital therapeutics, we must understand how people use technologies and then create solutions that fit their actual use patterns, not those that we expect them to do a priori. Focus groups can provide preliminary insights into questions of optimal engagement patterns, but further quantitative analysis is required to tease apart different subsets of effective and ineffective DMH intervention use.

Third, it is critical to observe which factors mediate engagement and develop microinterventions (eg, “nudge” notifications or design frictions) that can effectively target these factors. Finally, work could be done to extend the IE Model so that it can be validly applied to DMH contexts involving lay practitioners (eg, internet-based peer support groups). This latter research direction indicates the utility of holding additional exploratory focus groups in other DMH intervention contexts using the IE Model as a preliminary and nondefinitive conceptual guide. Together, these future lines of research point to the direction of a robust and empirically validated model of engagement and action in the guided DMH, which could allow intervention scientists to optimize user engagement, individualize intervention strategies, improve user experiences, and ultimately enhance mental health outcomes.

### Conclusions

In this study, we described our analysis of 5 digital therapy user focus groups. We synthesized our results using constructs from health science’s HAPA, design science’s LIM, and key interpersonal constructs from face-to-face psychotherapy research. We found that users’ self-reported engagement trajectories in digital therapy varied widely over the same platform but were principally informed by intrapsychic factors (eg, self-efficacy and outcome expectancy), interpersonal factors (eg, the therapeutic alliance and its rupture), and external factors (eg, treatment costs). We proposed a synthesis of these constructs in the IE Model of Digital Therapy and outlined the hypothesized connections between the constructs found in these 3 distinct research frameworks.

## Abbreviations

DMH: digital mental health
HAPA: Health Action Process Approach
IE: Integrative Engagement
LIM: Lived Informatics Model

## Data Availability

Owing to the sensitive nature of the personal information contained in the data set, access to the data underlying this study is restricted. The data set was obtained from a mental health platform and contains sensitive personal information about its users. Access to this data set is governed by strict ethical and legal regulations to protect the privacy and confidentiality of the individuals involved. Researchers who wish to access the data may do so by obtaining permission from the platform’s data access committee, subject to appropriate ethical and legal approvals. The deidentified data used in this study may be made available upon request to interested researchers who have obtained appropriate ethical and legal approvals from their own institutions and have signed a data use agreement with the platform. Requests for data access should be directed at derrick@talkspace.com.
